# Supplementary material for: A 4D‐STEM Tomographic Framework Assisted by Object Tracking for Nanoparticle Structure Determination
Source: Small Methods. 2026 Oct 7;10(21):e71095. doi: 10.1002/smtd.71095 (PMC13745972; doi:10.1002/smtd.71095)
Supplement: Supplementary file 1 [file SMTD-10-e71095-s002.docx]

**A 4D-STEM Tomographic Framework Assisted by Object Tracking for Nanoparticle Structure Determination**

*Saleh Gholam*, Arno Annys, Irina Skvortsova, Erica Cordero Oyonarte, Amirhossein Hajizadeh, Philippe Boullay, Johan Hofkens, Johan Verbeeck, Joke Hadermann**

S. G., A. A., I. S., A. H., J. V., J. Ha.

EMAT, Department of Physics, University of Antwerp, Groenenborgerlaan 171, Antwerp 2020, Belgium

E-mails: [saleh.gholam@gmail.com](mailto:saleh.gholam@gmail.com); [joke.hadermann@uantwerpen.be](mailto:joke.hadermann@uantwerpen.be)

E. C. O., P. B.

CRISMAT, Normandie Université, ENSICAEN, UNICAEN, CNRS UMR 6508, 6 Bd Maréchal Juin, F-14050 Cedex Caen, France

I. S., J. Ho.

Department of Chemistry, KU Leuven, Leuven 3001, Belgium

# 1. R-factor Definitions

The R-factors referred in the paper are calculated based on the following formula.

$$R_{int}=\frac{\sum_{hkl} \sum_{i} |I_{hkl,i}- \left\langle I_{hkl} \right\rangle|}{\sum_{hkl} \sum_{i} I_{hkl,i}}$$

(1)

$$R_{meas}=\frac{\sum_{hkl} \left[ \frac{n_{hkl}}{n_{hkl}-1} \right]^{\frac{1}{2}}\sum_{i} |I_{hkl,i}- \left\langle I_{hkl} \right\rangle}{\sum_{hkl} \sum_{i} I_{hkl,i}}$$

(2)

$$R_{obs}=\frac{\sum_{hkl} \left| \left| F_{o} \right|-\left| F_{c} \right| \right|}{\sum_{hkl} \left| F_{o} \right|}$$

(3)

In these equations, $I_{hkl,i}$ is the intensity of $i$-th observation for reflection $hkl$, $\left\langle I_{hkl} \right\rangle$is the mean intensity of all symmetry-equivalent observations for reflection $hkl$, $n_{hkl}$ number of symmetry-equivalent observations of reflection $hkl$, and $F_{o}$ and $F_{c}$ are respectively observed and calculated structure factor amplitudes.

# 2. Threshold for Skipping Reflections

Omitting reflections is a sensitive subject in crystallographic studies for structure solution, as this can force the model toward a specific direction against observations. However, coincidence losses and localized pixel saturations occur on detectors with Timepix3 chips at high fluxes.^[1]^ These introduce direct errors on the integration process. This is highly likely in parallel beam illumination as the collected signal is localized at Bragg spots. Although it is attempted to reject these errors for the kinematical refinement using error-model adjustments implemented in PETS2 software ^[2]^, the dynamical refinement uses intensities of reflections at each frame to form virtual frames ^[3]^, and consequently such counting errors will be included. Omission of the reflections are performed here when the model is refined and stabilized after an initial dynamical refinement. It is essential to note that the distinction of the clipping effect is not trivial as it does not occur at a fixed value for the event-based detectors. Additionally, it is even more complex to measure this for the extracted frames from 4D-STEM tomography, since frames are the result of summing a varying region on 4D-STEM tomography data and at different crystal orientations.

To select an effective and yet a safe threshold for omitting reflections during dynamical refinement, a range of values were checked during the dynamical refinement of T-P1 and T-P2 particles. In Jana software, the threshold is based on the following equation:

| \|F_obs_ – F_calc_\| > threshold × sig(F_obs_) | (4) |
| --- | --- |
|  |  |

**Figure S 1** demonstrates the results after tweaking this threshold in a wide range and its influence on the number of reflections which are skipped, dynamical refinement R_obs_ and the largest residual charge in the difference Fourier maps. Both particles show a sudden decrease in R_obs_ in the range between 20 and 30. A closer look at the reflection profiles and 3D ED frames showed most of the reflections culled in this range are strong reflections close to a zone axis, and some of them even have saturated intensities in their intensity profile. Thus, it is highly likely that these reflections suffer from both the detector non-linearities and heavy

dynamical effects. The convolution of these effects can cause deviation in the dynamical refinement. Threshold 25 was selected as a more conservative threshold for 3D ED datasets extracted from DS-1 dataset. The threshold was lowered to 20 for DS-3 dataset with CsPbBr_3_.

**Figure S 1**. The influence of the threshold for skipping reflections on the dynamical refinement of the brookite structure; (a) T-P1, (b) T-P2

# 3. Probe Size in STEM

The achievable probe size can be estimated by the squared contribution of the beam spreading effects. ^[4, 5]^ Assuming Gaussian shape and independence of the these effects, the probe size is influenced by the contributions from the initial Gaussian diameter at the gun (d_g_), diffraction at the final aperture (d_d_), the spherical aberration effects in the beam-forming lens (d_s_), and the chromatic aberration blurring (d_c_). For a TEM without aberration-correction, these can be estimated as:

$$d_{g}=\frac{2}{\pi\alpha} \sqrt{\frac{I_{p}}{\beta}}$$

(5)

$$d_{d}=\frac{0.61\lambda}{\alpha}$$

(6)

$$d_{s}=0.5C_{s}\alpha^{3}$$

(7)

$$d_{c}=C_{c}\left( \frac{\Delta E}{E_{0}} \right)$$

(8)

In these equations, $\alpha$, $\beta$, and $I_{p}$ respectively stand for the convergence angle, the brightness of the source and the probe current, $\lambda$ is the wavelength of the accelerated electrons, $C_{s}$ is the spherical aberration coefficient, and $C_{c}$, $\Delta E$ and $E_{0}$ are respectively the chromatic aberration coefficient, the energy spread of the electron beam and the energy of the electrons. Then, the final probe size can be calculated as:

$$d_{probe}=\sqrt{d_{g}^{2}+d_{d}^{2}+d_{s}^{2}+d_{c}^{2}}$$

(9)

The assumptions for the calculation of the probe size are listed in **Table S 1**. For achieving the probe FWHM according to Kirkland ^[6]^, the prefactors of the abovementioned components should be respectively changed to 0.514, 0.18, 0.6 and 0.707.

**Table S 1**. The estimated microscope parameters used for the theoretical probe size calculations.

| $\boldsymbol{\beta}\left[ \boldsymbol{A.}\boldsymbol{m}^{\boldsymbol{-2}}\boldsymbol{s}\boldsymbol{r}^{\boldsymbol{-1}} \right]$ | $\boldsymbol{I}_{\boldsymbol{p}} \left[ \boldsymbol{A} \right]$ | $\boldsymbol{\lambda[pm]}$ | $\boldsymbol{C}_{\boldsymbol{s}} \left[ \boldsymbol{mm} \right]$ | $\boldsymbol{C}_{\boldsymbol{c}} \left[ \boldsymbol{mm} \right]$ | $\boldsymbol{\Delta}\boldsymbol{E}\left[ \boldsymbol{eV} \right]$ | $\boldsymbol{E}_{\boldsymbol{0}} \left[ \boldsymbol{eV} \right]$ |
| --- | --- | --- | --- | --- | --- | --- |
| $\boldsymbol{5\times}\boldsymbol{10}^{\boldsymbol{12}}$ | $5\times{10}^{-12}$ | 2.508 | 1.5 | 1.5 | 0.5 | 200,000 |

# 4. Disk Overlaps in Diffraction

As the convergence of the electron beam increases, the size of the reflections increases in the reciprocal space. To be able to follow 3D ED data analysis, the disk overlaps should be avoided. On one hand, there is a relation between the size of the disks and the convergence angle. On the other hand, the distance between the disks depends on the size of the unit cell and the orientation of the crystal. Thus, there is a relation between the size of the unit cell and the largest convergence angle which can be selected. As illustrated in **Figure S2**(**a)**, by trigonometry $D_{disk}=2\alpha/\lambda$. In the reciprocal space, the minimum distance between the disks are when the crystal orients along the maximum interplanar spacing (d-spacing) of the structure. Therefore, the condition $D_{disk}<\frac{1}{d-spacing^{max}}$ is the key for avoiding disk overlaps. Thus, for studying a structure, d-spacing should be smaller than $\lambda/2\alpha$. For an orthorhombic structure (such as the samples studied in this work), largest d-spacing equals to the maximum of the unit cell parameters a, b and c. With respect to the probable charge spreading of the incoming electrons in the detector, it would be safer to keep the convergence somewhat smaller than such ideal calculation so that there would be no chance of disk overlaps. It should be noted that these limitations are relaxed at lower acceleration voltages as they have higher wavelengths (**Figure S 2b**).

**Figure S 2**. (a) The relationship between the convergence angle and the overlap of the reflection disks; (b) the limits of d-spacing for not having disk overlap at different acceleration voltages.

# 5. Supplementary Figures

**Figure S 3**. The probe image formed using (a) 20 µm aperture and (b) 10 µm aperture with their Gaussian fit for FWHM calculations in (b, e) vertical and (c, f) horizontal directions. The average of the horizontal and vertical values are reported in the main text. The images were acquired by a US1000 CCD detector (Gatan).

**Figure S 4**. An example of blurring and binarization on a tracked ROI on C-P2.

**Figure S 5**. The presence of two different domains in T-P5, i.e. D1 and D2; (a) overlay of the integration masks for D1 and D2 in the 3D ED dataset, (b) selected reflections as virtual detectors for visualizing D1 and D2, (c) the overlay of the domain masks over the particle.

**Figure S 6**. Brookite atomic structure in (a) ball and stick model, (b) polyhedral model and (c) with 50% probability for atomic movements. The structure is based on T-P4 after dynamical refinement with culled reflections.

**Figure S 7**. CsPbBr_3_ atomic structure from different view axes. The tilted polyhedra can be observed in both views. The model is based on C-P1 after dynamical refinement with culled reflections.

**Figure S 8**. Diffraction patterns at α = -50̊ for (a) T-P1, (b) T-P1-E1 and (c) T-P1-E2.

**Figure S 9.** Segmentation and tracking using SAM2 tracker on the agglomerate of some particles in DS-3 dataset.

# 6. Supplementary Tables

In the upcoming tables, the following terms are used:

- Nall: Total number of unique reflections
- Nobs: Number of unique, observed reflections
- Completeness: the percentage of observed reflections by considering the symmetry of the system, showing the coverage of the reciprocal space
- Redundancy: The average number of times a reflection is observed in the dataset
- <I/σ>: The average signal-to-noise ration of the measured intensities
- wR(obs): Weighted R-factors during refinement calculated on observed reflections

**Table S 2**. Data reduction statistics for 3D-ED datasets extracted from TiO_2_ particles in dataset DS-1.

|  | | T-P1 | T-P2 | T-P3 | T-P4 | T-P1-E1 | T-P1-E2 |
| --- | --- | --- | --- | --- | --- | --- | --- |
| Tilt Range [ ̊ ] | | [-50, 30] | [-50, 28] | [-50, 30] | [-50, 30] | [-50, 30] | [-50, 30] |
| Res. [Å^-1^] | | 2.0 | 2.0 | 2.0 | 2.0 | 2.0 | 2.0 |
| Found Unit Cell^a^ | a [Å],  b [Å],  c [Å] | 9.157(2)  5.469(0)  5.075(0) | 9.144(3)  5.397(1)  5.157(0) | 9.200(1)  5.445(1)  5.075(0) | 9.159(3)  5.433(1)  5.116(0) | 9.178(7)  5.471(1)  5.073(1) | 9.168(4)  5.469(1)  5.072(0) |
|  | α [º],  β [º],  γ [º] | 90.06(1)  89.80(1)  90.29(1) | 89.43(1)  89.85(1)  90.47(2) | 89.79(0)  89.94(1)  90.35(1) | 90.87(1)  89.50(1)  89.93(3) | 90.00(2)  89.70(3)  90.19(4) | 89.96(1)  89.77(1)  89.77(2) |
| Nobs / Nall | | 269 / 921 | 504 / 1014 | 298 / 998 | 583 / 1013 | 240 / 927 | 293 / 922 |
| Redundancy | | 4.16 | 3.64 | 3.80 | 3.77 | 4.14 | 4.15 |
| Completeness [%] | | 71.8 | 79.1 | 77.9 | 79.0 | 72.3 | 71.9 |
| <I/σ> | | 3.45 | 4.31 | 3.79 | 4.93 | 3.48 | 3.52 |
| R_int_ (obs) [%] | | 16.37 | 10.85 | 12.71 | 9.10 | 11.17 | 14.25 |
| R_meas_ (obs) [%] | | 18.94 | 12.80 | 14.90 | 10.54 | 12.99 | 16.44 |

^a^ The reported unit cell is the raw cell found in the data. Then, the distortion refinements were performed using the fixed cell method reported in the previous work based on synchrotron XRD results. ^[6]^

^b^ As the data is step-wise, and not continuous, PETS2 calculates high partialities with respect to the lost data between the collected points.

**Table S 3**. Structure refinement statistics for the TiO_2_ particles in dataset DS-1.

| Type | Parameter | T-P1 | T-P2 | T-P3 | T-P4 | T-P1-E1 | T-P1-E2 |
| --- | --- | --- | --- | --- | --- | --- | --- |
| Kinematical | # Ref. / # Param. | 591 / 13 | 725 / 13 | 562 / 13 | 755 / 13 | 506 / 13 | 599 / 13 |
|  | Goodness of Fit | 3.81 | 3.61 | 3.02 | 4.09 | 2.48 | 3.23 |
|  | R(obs) [%] | 26.28 | 21.50 | 19.35 | 20.46 | 16.42 | 21.77 |
|  | wR(obs) [%] | 30.22 | 25.64 | 23.58 | 25.68 | 20.06 | 25.91 |
|  | Avg. Distance [Å] | 0.018 | 0.014 | 0.015 | 0.012 | 0.013 | 0.016 |
|  | Max Distance [Å] | 0.027 | 0.026 | 0.028 | 0.018 | 0.024 | 0.026 |
| Kinematical with Extinction Correction | # Ref. / # Param. | - | 725 / 14 | 562 / 14 | - | 506 / 14 | 599 / 14 |
|  | Goodness of Fit | - | 3.37 | 2.57 | - | 2.39 | 2.92 |
|  | R(obs) [%] | - | 18.40 | 13.65 | - | 14.22 | 18.76 |
|  | wR(obs) [%] | - | 23.94 | 20.06 | - | 19.34 | 23.42 |
|  | Avg. Distance [Å] | - | 0.013 | 0.014 | - | 0.013 | 0.016 |
|  | Max Distance [Å] | - | 0.026 | 0.020 | - | 0.023 | 0.025 |
| Dynamical | # Ref. / # Param. | 3243 / 56 | 2686 / 55 | 2517 / 56 | 2640 / 56 | 1697 / 56 | 2683 / 56 |
|  | Goodness of Fit | 7.12 | 5.65 | 5.19 | 5.81 | 2.91 | 4.86 |
|  | R(obs) [%] | 12.15 | 11.69 | 11.44 | 12.68 | 9.97 | 10.92 |
|  | wR(obs) [%] | 14.30 | 13.73 | 13.53 | 14.69 | 10.41 | 12.76 |
|  | Avg. Distance [Å] | 0.006 | 0.005 | 0.007 | 0.005 | 0.010 | 0.008 |
|  | Max Distance [Å] | 0.009 | 0.006 | 0.008 | 0.006 | 0.018 | 0.015 |
| Dynamical with Skipping Reflections | # Ref. / # Param. | 3194 / 56 | 2686 / 55 | 2493 / 56 | 2614 / 56 | 1697 / 56 | 2667 / 56 |
|  | # Culled Ref. | 49 | 21 | 24 | 26 | 0 | 4.05 |
|  | Goodness of Fit | 5.33 | 4.22 | 3.93 | 4.66 | 2.91 | 16 |
|  | R(obs) [%] | 9.63 | 9.41 | 9.32 | 11.16 | 9.97 | 9.63 |
|  | wR(obs) [%] | 10.91 | 10.42 | 10.46 | 12.06 | 10.41 | 10.78 |
|  | Avg. Distance [Å] | 0.006 | 0.007 | 0.008 | 0.006 | 0.010 | 0.008 |
|  | Max Distance [Å] | 0.011 | 0.010 | 0.010 | 0.008 | 0.018 | 0.015 |
| Dynamical with Anisotropic ADP | # Ref. / # Param. | 3194 / 71 | 2665 / 70 | 2494 / 71 | 2614 / 71 | 1697 / 71 | 2666 / 71 |
|  | # Culled Ref. | 49 | 21 | 23 | 26 | 0 | 17 |
|  | Goodness of Fit | 5.22 | 4.15 | 3.92 | 4.62 | 2.88 | 3.97 |
|  | R(obs) [%] | 9.42 | 9.24 | 9.22 | 11.02 | 9.85 | 9.41 |
|  | wR(obs) [%] | 10.66 | 10.23 | 10.41 | 11.93 | 10.23 | 10.54 |
|  | Avg. Distance [Å] | 0.006 | 0.006 | 0.008 | 0.006 | 0.010 | 0.008 |
|  | Max Distance [Å] | 0.010 | 0.009 | 0.009 | 0.007 | 0.014 | 0.014 |

**Table S 4**. Bond Valence Sum (BVS) values of TiO_2_ atoms for the solved structures in datasets DS-1. The BVS of the reference structure (ICSD ID 154605) was 4.025(17), 2.074(13) and 1.951(11) for respectively Ti 1, O 1 and O2 atoms.

|  |  | T-P1 | T-P2 | T-P3 | T-P4 | T-P1-E1 | T-P1-E2 |
| --- | --- | --- | --- | --- | --- | --- | --- |
| Kinematical | Ti 1 | 4.02(3) | 4.03(2) | 4.01(3) | 4.02(2) | 4.02(3) | 4.02(3) |
|  | O 1 | 2.07(2) | 1.97(2) | 2.04(2) | 2.07(2) | 2.06(2) | 2.06(2) |
|  | O 2 | 1.96(2) | 2.06(2) | 1.97(2) | 1.96(1) | 1.96(2) | 1.96(2) |
| Kinematical with Extinction Correction | Ti 1 | - | 4.03(2) | 4.01(2) | - | 4.02(3) | 4.02(2) |
|  | O 1 | - | 1.95(1) | 2.05(2) | - | 2.06(2) | 2.07(2) |
|  | O 2 | - | 2.08(2) | 1.96(2) | - | 1.96(2) | 1.96(2) |
| Dynamical | Ti 1 | 4.02(1) | 4.02(1) | 4.02(1) | 4.02(1) | 4.02(2) | 4.02(1) |
|  | O 1 | 2.058(8) | 1.958(9) | 2.060(9) | 2.07(1) | 2.05(1) | 2.056(9) |
|  | O 2 | 1.959(7) | 2.06(1) | 1.958(8) | 1.953(9) | 1.97(1) | 1.960(8) |
| Dynamical with Skipped Reflections | Ti 1 | 4.020(7) | 4.025(9) | 4.020(9) | 4.02(1) | 4.02(2) | 4.018(8) |
|  | O 1 | 2.058(5) | 1.955(6) | 2.064(7) | 2.070(7) | 2.06(1) | 2.068(6) |
|  | O 2 | 1.961(5) | 2.070(7) | 1.956(6) | 1.950(6) | 1.97(1) | 1.950(6) |
| Dynamical with Anisotropic ADP | Ti 1 | 4.019(7) | 4.026(9) | 4.020(9) | 4.02(1) | 4.02(2) | 4.018(8) |
|  | O 1 | 2.058(5) | 1.956(6) | 2.064(7) | 2.070(7) | 2.06(1) | 2.068(6) |
|  | O 2 | 1.961(5) | 2.070(7) | 1.956(6) | 1.951(6) | 1.97(1) | 1.950(5) |

**Table S 5**. Data reduction statistics for 3D-ED datasets extracted from CsPbBr_3_ particles in dataset DS-3.

| Particle Code | | C-P1 | C-P2 | C-P3 |
| --- | --- | --- | --- | --- |
| Tilt Range [ ̊ ] | | [-35, 34] | [-35, 35] | [-35, 35] |
| Resolution [Å^-1^] | | 1.4 | 1.4 | 1.4 |
| Found Unit Cell | a [Å] | 8.296(2) | 8.270(1) | 8.239(3) |
|  | b [Å] | 8.357(2) | 8.258(2) | 8.353(4) |
|  | c [Å] | 11.738(3) | 11.846(5) | 11.788(7) |
|  | α [º] | 90.18(2) | 90.40(4) | 89.03(6) |
|  | β [º] | 90.51(0) | 91.00(3) | 89.55(6) |
|  | γ [º] | 90.86(0) | 89.34(3) | 89.78(4) |
| Refined Unit Cell^a^ | a [Å] | 8.290(3) | 8.280(1) | 8.323(4) |
|  | b [Å] | 8.326(3) | 8.328(1) | 8.304(4) |
|  | c [Å] | 11.819(4) | 11.861(2) | 11.831(5) |
|  | α [º] | 90(0) | 90(0) | 90(0) |
|  | β [º] | 90(0) | 90(0) | 90(0) |
|  | γ [º] | 90(0) | 90(0) | 90(0) |
| Nobs / Nall | | 379 / 1166 | 340 / 1056 | 364 / 1160 |
| Redundancy | | 2.94 | 3.28 | 2.98 |
| Completeness [%] | | 84.1 | 75.9 | 83.5 |
| <I/σ> | | 4.29 | 4.23 | 4.30 |
| R_int_ (obs) [%] | | 9.35 | 8.97 | 10.63 |
| R_meas_ (obs) [%] | | 11.44 | 10.83 | 12.82 |

^a^ The refined unit cells are after distortion refinement by assuming orthorhombic symmetry.

**Table S 6**. Structure refinement statistics for the CsPbBr_3_ particles in dataset DS-3.

| Refinement Type | Parameter | C-P1 | C-P2 | C-P3 |
| --- | --- | --- | --- | --- |
| Kinematical | # Reflection / # Parameter | 530 / 12 | 502 / 12 | 495 / 12 |
|  | Goodness of Fit | 3.10 | 3.59 | 3.86 |
|  | R(obs) [%] | 17.87 | 18.75 | 19.64 |
|  | wR(obs) [%] | 24.16 | 27.31 | 28.55 |
| Kinematical with Extinction Correction | # Reflection / # Parameter | 530 / 13 | 502 / 13 | 495 / 13 |
|  | Goodness of Fit | 3.09 | 3.59 | 3.86 |
|  | R(obs) [%] | 17.87 | 18.67 | 19.67 |
|  | wR(obs) [%] | 24.11 | 27.30 | 28.55 |
| Dynamical | # Reflection / # Parameter | 1007 / 50 | 1240 / 51 | 962 / 51 |
|  | Goodness of Fit | 4.00 | 3.28 | 3.68 |
|  | R(obs) [%] | 12.83 | 10.64 | 12.18 |
|  | wR(obs) [%] | 14.46 | 11.57 | 12.98 |
| Dynamical with Skipping Reflections | # Reflection / # Parameter | 1002 / 50 | 1240 / 51 | 961 / 51 |
|  | # Culled Reflection | 5 | 0 | 1 |
|  | Goodness of Fit | 3.64 | 3.28 | 3.59 |
|  | R(obs) [%] | 12.34 | 10.64 | 12.04 |
|  | wR(obs) [%] | 13.27 | 11.57 | 12.65 |
| Dynamical with Anisotropic ADP | # Reflection / # Parameter | 1003 / 66 | 1240 / 67 | 961 / 67 |
|  | # Culled Reflection | 4 | 0 | 1 |
|  | Goodness of Fit | 2.90 | 2.64 | 2.79 |
|  | R(obs) [%] | 9.40 | 8.13 | 9.09 |
|  | wR(obs) [%] | 10.43 | 9.26 | 9.75 |

**Table S 7**. Bond Valence Sum (BVS) values of CsPbBr_3_ atoms for the solved structures in datasets DS-3.

| Refinement | Atom | C-P1 | C-P2 | C-P3 |
| --- | --- | --- | --- | --- |
| Kinematical | Pb 1 | 2.38(2) | 2.36(2) | 2.42(3) |
|  | Cs 1 | 0.63(1) | 0.65(1) | 0.62(1) |
|  | Br 1 | 1.00(1) | 0.98(1) | 1.02(2) |
|  | Br 2 | 1.00(1) | 1.01(2) | 1.01(1) |
| Kinematical with Extinction Correction | Pb 1 | 2.39(2) | 2.36(2) | 2.42(3) |
|  | Cs 1 | 0.63(1) | 0.65(1) | 0.66(1) |
|  | Br 1 | 1.00(1) | 0.98(1) | 1.02(2) |
|  | Br 2 | 1.00(1) | 1.01(2) | 1.04(1) |
| Dynamical | Pb 1 | 2.39(2) | 2.35(1) | 2.41(2) |
|  | Cs 1 | 0.63(1) | 0.63(0) | 0.60(1) |
|  | Br 1 | 1.00(1) | 0.98(0) | 1.01(1) |
|  | Br 2 | 1.00(1) | 1..00(1) | 1.00(1) |
| Dynamical with Skipped Reflections | Pb 1 | 2.39(2) | 2.35(1) | 2.42(1) |
|  | Cs 1 | 0.63(1) | 0.63(0) | 0.59(1) |
|  | Br 1 | 1.00(1) | 0.98(0) | 1.01(1) |
|  | Br 2 | 1.00(1) | 1.00(1) | 1.00(1) |
| Dynamical with Anisotropic ADP | Pb 1 | 2.38(1) | 2.35(1) | 2.42(1) |
|  | Cs 1 | 0.63(1) | 0.63(4) | 0.59(1) |
|  | Br 1 | 1.00(1) | 0.98(0) | 1.01(1) |
|  | Br 2 | 1.00(1) | 1.00(1) | 0.99(1) |

# 7. Supplementary Files

The CIF file for all structures solved in the paper is in the supplementary files. The name of the files is for instance as TP1_02_kin, standing for TP-1 particle after kinematical refinement. The refinement stages are denoted as follows in the names:

- Kinematical refinement: kin
- Kinematical refinement with extinction correction: exti
- Dynamical refinement: dyn
- Dynamical refinement with culled reflections: cull
- Dynamical refinement with culled reflections and anisotropic ADP: aniso

# 8. References

1. Annys, A., et al., Removing constraints of 4D-STEM with a framework for event-driven acquisition and processing. Ultramicroscopy, 2025. **277**: p. 114206.

2. Khouchen, M., et al., Optimal estimated standard uncertainties of reflection intensities for kinematical refinement from 3D electron diffraction data. Acta Crystallographica Section A, 2023. **79**(5): p. 427-439.

3. Klar, P.B., et al., Accurate structure models and absolute configuration determination using dynamical effects in continuous-rotation 3D electron diffraction data. Nature Chemistry, 2023. **15**(6): p. 848-855.

4. Kohl, H. and L. Reimer, Transmission Electron Microscopy. Springer Series in Optical Sciences. Vol. 36. 2008.

5. Williams, D.B. and C.B. Carter, The transmission electron microscope, in Transmission electron microscopy: a textbook for materials science. 1996, Springer. p. 3-17, 215, 274.

6. Kirkland, E.J., Advanced computing in electron microscopy. 3 ed. Vol. 12. 2020: Springer.
